# A Qualitative Comparison of the Reactivities of 3,4,4,5-Tetrachloro-4*H*-1,2,6-thiadiazine and 4,5-Dichloro-1,2,3-dithiazolium Chloride

**DOI:** 10.3390/molecules200814576

**Published:** 2015-08-12

**Authors:** Andreas S. Kalogirou, Panayiotis A. Koutentis

**Affiliations:** Department of Chemistry, University of Cyprus, P.O. Box 20537, Nicosia 1678, Cyprus; E-Mail: koutenti@ucy.ac.cy

**Keywords:** heterocycle, 1,2,6-thiadiazines, Appel’s salt, 1,2,3-dithiazoles

## Abstract

The high yielding transformations of 3,4,4,5-tetrachloro-4*H*-1,2,6-thiadiazine into 3,5-dichloro-4*H*-1,2,6-thiadiazin-4-one (up to 85%) and 2-(3,5-dichloro-4*H*-1,2,6-thiadiazin-4-ylidene)malononitrile (up to 83%) have been investigated and compared to the analogous transformations of the closely-related 4,5-dichloro-1,2,3-dithiazolium chloride (Appel’s salt) into 4-chloro-5*H*-1,2,3-dithiazol-5-one and 2-(4-chloro-5*H*-1,2,3-dithiazol-5-ylidene)malononitrile. Furthermore, cyclocondensation of 3,4,4,5-tetrachloro-4*H*-1,2,6-thiadiazine with 2-aminophenol and 1,2-benzenediamines gave fused 4*H*-1,2,6-thiadiazines in 68%–85% yields.

## 1. Introduction

**Scheme 1:**
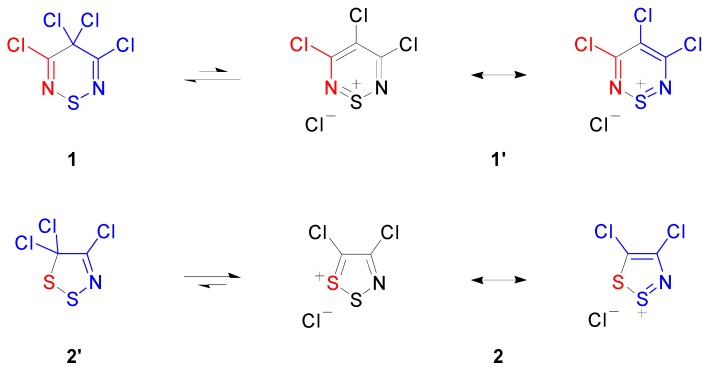
Comparison of 3,4,4,5-tetrachloro-4*H*-1,2,6-thiadiazine (**1**) with 4,5-dichloro-1,2,3-dithiazolium chloride (**2**).

3,4,4,5-Tetrachloro-4*H*-1,2,6-thiadiazine (**1**), which possesses a reactive geminal dichloromethylene, is related to highly-electrophilic 4,5-dichloro-1,2,3-dithiazolium chloride (**2**) (Appel’s salt), in that substitution of the S-1 sulfur by a Cl–C=N unit affords 3,4,5-trichloro-1,2,6-thiadiazinium chloride (**1′**), the ionic form of **1** (Scheme 1). The late C. W. Rees recognized that the structural and electronic similarities implied that the C-4 position of tetrachlorothiadiazine **1** and the C-5 position of Appel’s salt **2** could have similar chemical reactivities. Both of these compounds are useful and attractive heterocycles; however, considerably more work has been reported for Appel’s salt **2**, presumably owing to its more facile synthesis.

Non-oxidized 4*H*-1,2,6-thiadiazines are rare, and accounts of their chemistry and applications are few. Mono-5-substituted-4*H*-1,2,6-thiadiazines have been investigated as plant antifungals [1,2,3,4,5], and to the best of our knowledge, these studies were not expanded. Furthermore, fused analogues were studied as examples of “extreme quinoids” that have an ambiguous aromatic character [6], while others displayed unusual liquid crystalline properties or behaved as near-infrared dyes [7,8]. Moreover, selected 4*H*-1,2,6-thiadiazines have been proposed as radical anion precursors for molecule-based magnetic and conducting functional materials [9]. 4*H*-1,2,6-Thiadiazin-4-one containing small molecules have been investigated as efficient electron donors in solution-processed bulk heterojunction solar cells [10], while in an effort to understand their optical properties, key 4*H*-1,2,6-thiadiazines have been characterized by resonance Raman (RR), absorption (UV-VIS) and photoluminescence (PL) spectroscopies [11]. For these potential applications to become reality, the chemistry of 4*H*-1,2,6-thiadiazines needs to be further developed. To date, the most commonly used non-*S*-oxidized 4*H*-1,2,6-thiadiazine scaffold is 3,5-dichloro-4*H*-1,2,6-thiadiazin-4-one (**3**), which is a precursor to many thiadiazine derivatives, via palladium-catalyzed C-C coupling reactions, such as Stille and Suzuki-Miyaura couplings, to give various 3,5-di(het)aryl substituted systems [12,13,14] and reactions with amines to give mono-, or 3,5-diamino-substituted systems [15], or polycyclic systems [16].

Appel’s salt **2**, discovered in 1985, is readily prepared from chloroacetonitrile and disulfur dichloride [17] and has since found numerous uses as a scaffold for the synthesis of 1,2,3-dithiazole derivatives [18,19,20,21]. Dithiazolium **2** exists as a salt and not in the covalent form **2′**, and it is a planar and 6π aromatic system, although worthy of note is the 5,5-difluoro analogue, which is a non-ionic covalent bound molecule that can be distilled and isolated as an oil [17]. The chemistry of the dithiazolium **2** is governed by the electrophilicity of the C-5 carbon, and it readily reacts with nucleophiles to give neutral 5*H*-1,2,3-dithiazoles.

**Scheme 2:**
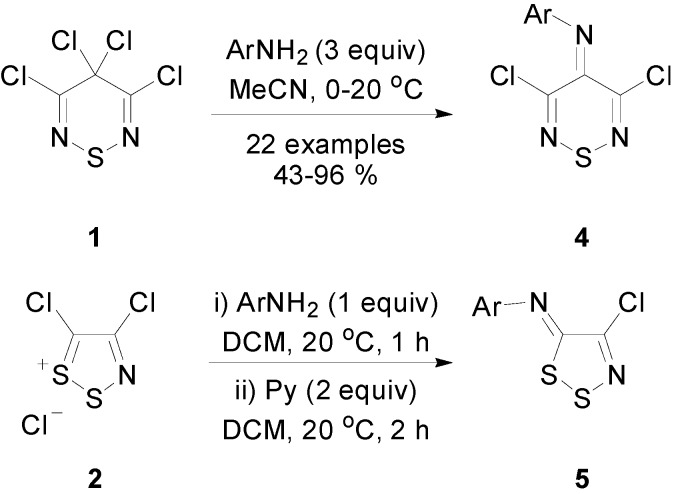
Synthesis of synthesis of *N*-aryl-3,5-dichloro-4*H*-1,2,6-thiadiazin-4-imines (**4**) and (*Z*)-*N*-aryl-4-chloro-5*H*-1,2,3-dithiazol-5-imines (**5**).

Bearing in mind the similarities of the two reagents thiadiazine **1** and Appel’s salt **2**, we began an investigation to compare their reactions with simple nucleophiles. Recently, the synthesis of *N*-aryl-3,5-dichloro-4*H*-1,2,6-thiadiazin-4-imines (**4**) from 3,4,4,5-tetrachloro-4*H*-1,2,6-thiadiazine (**1**) [22], by a simple reaction with aniline was investigated. This condensation is very similar to the reaction of anilines with Appel’s salt **2** that gives (*Z*)-*N*-aryl-4-chloro-5*H*-1,2,3-dithiazol-5-imines **5** (Scheme 2) [23]. We now report the reactivity of thiadiazine **1** with three different types of nucleophiles: water, malononitrile and aromatic bisnucleophiles.

## 2. Results and Discussion

### 2.1. Preparation of 3,4,4,5-Tetrachloro-4H-1,2,6-thiadiazine (***1***)

**Scheme 3:**
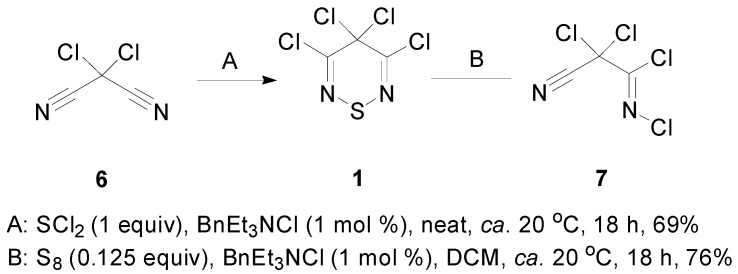
Synthesis of 3,4,4,5-tetrachloro-4*H*-1,2,6-thiadiazine (**1**).

Two methods exist in the literature for the preparation of the tetrachlorothiadiazine **1**: the reaction of dichloromalononitrile (**6**) and SCl_2_ [15] and the reaction of *N*-2,2-trichloro-2-cyanoacetimidoyl chloride (**7**) [24] with elemental sulfur. In our hands, both reactions worked well; however, the second method was preferred, as it gave better yields and avoided the use of the toxic and hard to access SCl_2_ (Scheme 3). Compound **7**, even though it looks very reactive, was easy to prepare and isolate and can be stored for up to six months at 0 °C. Moreover, the two reports on the isolation of tetrachlorothiadiazine **1** gave conflicting distillation data for the product (100 °C, 8 mbar [25] *vs.* 90 °C, 4 mbar [26]). In our hands, the thiadiazine **1** distilled nicely at (90 °C, 30 mbar) as a pale yellow oil that crystallized on cooling to −20 °C [mp (DSC) onset: 10.3 °C, peak max: 12.8 °C] and can be stored for several months at −40 °C.

**Scheme 4:**
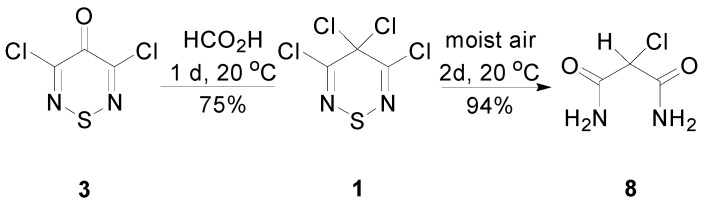
Known reactions of 3,4,4,5-tetrachloro-4*H*-1,2,6-thiadiazine (**1**).

3,5-Dichloro-4*H*-1,2,6-thiadiazin-4-one (**3**) can be prepared from the reaction of glacial formic acid with 3,4,4,5-tetrachloro-4*H*-1,2,6-thiadiazine (**1**) [15]. In our hands, this transformation was sensitive to the quality of the formic acid used as both a solvent and reagent. Interestingly, no other chemistry has been reported for tetrachlorothiadiazine **1** apart from this reaction and its degradation in moist air to give 2-chloromalonamide (**8**) (Scheme 4) [26]. As such, we reinvestigated the transformation of tetrachlorothiadiazine **1** into the thiadiazinone **3** (Section 2.2.).

Comparing the syntheses of tetrachlorothiadiazine **1** with dithiazolium **2**, it was clear that both required similarly hazardous reagents for their preparations. The synthesis of dithiazolium **2**, however, was clearly easier, as it required only commercially-available chloroacetonitrile and disulfur dichloride and can be isolated as a salt by simple filtration. On the other hand, the synthesis of the thiadiazine **1** required the use of chlorine gas to prepare the less readily-available dichloromalononitrile and SCl_2_ reagents, and the product **1** was isolated by vacuum distillation.

### 2.2. Preparation of the Thiadiazinone ***3*** and Dithiazolone ***9***

Both the covalent thiadiazine **1** and the ionic 1,2,3-dithiazolium **2** are hydrolytically unstable, reacting with water or even in moist air. However, while the degradation of dithiazolium **2** in moist air gives a brown mass from which the dithiazolone **9** can be isolated by sublimation [17], the analogous degradation of tetrachlorothiadiazine **1** leads to ring cleavage to afford 2-chloromalonamide (**8**) (Scheme 4) [26].

Several improved methods are known for the transformation of Appel’s salt **2** to dithiazolone **9** [17,27], but only details of the formic acid-induced reaction have been reported for the hydrolysis of the tetrachlorothiadiazine **1** [1,2,15]. As such, we investigated other routes to synthesize the two ketones **3** and **9**.

The hydrolytic conditions investigated included treatment with formic acid, acetic acid, sodium and silver nitrates and DMSO (Table 1). The highest yielding (85%) and cleanest reaction conditions for the hydrolysis of the tetrachlorothiadiazine **1** into the thiadiazinone **3** involved the use of AgNO_3_ in MeCN (Table 1, Entry 6), while for the hydrolysis of Appel’s salt **2** to dithiazolone **9**, the cleanest conditions were using neat glacial formic acid (Table 1, Entry 1; 89%). While most procedures worked equally well for both transformations, significant differences in behavior were also observed.

The reaction of tetrachlorothiadiazine **1** with glacial formic acid (98% purity) gave the thiadiazinone **3** in a reasonable 75% yield, but the use of technical-grade formic acid (85% purity), containing *ca.* 15% water, led to degradation of the starting material, affording only a trace of 2-chloromalonamide (<1%). In contrast, Appel’s salt **2** was less sensitive to the presence of water in the formic acid and with the respective reactions gave dithiazolone **9** in 89% and 71% yields (Table 1, Entries 1 and 2).

Moreover, the classical reaction of Appel’s salt **2** with NaNO_3_ (1 equiv) in dichloromethane (DCM), at *ca.* 20 °C, to give dithiazolone **9** in a 72% yield [17] failed to give any product with the tetrachlorothiadiazine **1**; however, when DCM was replaced by MeCN, the desired thiadiazinone **3** was obtained in a 71%–72% yield (Table 1, Entries 4 and 5). Presumably, the more polar MeCN encouraged the equilibrium between the covalent and ionic forms of thiadiazine **1** to shift favorably towards the latter.

**Table 1:** Hydrolysis of tetrachlorothiadiazine **1** and Appel’s salt **2** to thiadiazinone **3** and dithiazolone **9**, respectively. 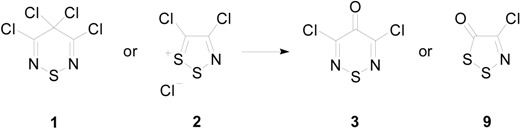

Entry	Conditions *^a^*	Product Yields and Reaction Times	
1	HCO_2_H (98%) neat	**3** (75%), 24 h	**9** (89%), 2 h
2	HCO_2_H (85%) *^b^* neat	**3** (0%), *^c^* 18 h	**9** (72%), 1 h
3	AcOH neat	**3** (74%), 48 h	**9** (60%), 2 h
4	NaNO_3_ (1 equiv), DCM	**3** (nr), *^d^* 18 h	**9** (72%), 18 h
5	NaNO_3_ (1 equiv), MeCN	**3** (71%), 1.5 h	**9** (77%), 18 h
6	AgNO_3_ (1 equiv), MeCN	**3** (85%), 0.5 h	**9** (72%), 1 min
7	Ag_2_SO_4_ (0.5 equiv), MeCN	**3** (77%), 4 h	**9** (73%), 24 h
8	H_2_O (1 equiv), DMSO (1 mol %), MeCN	**3** (0%), *^c^* 20 h	**9** (86%), 1 h
9	DMSO (1 equiv), MeCN	**3** (31%), 20 h	**9** (83%), 1.5 h
10	DMSO neat	**3** (45%), 1 h	**9** (50%), 15 min

*^a^* All reactions were performed at 20 °C. *^b^* Technical grade formic acid, contains 15% H_2_O. *^c^* Degradation to chloromalonamide **8**. *^d^* nr = no reaction.

Finally, Appel’s salt **2** can react with H_2_O (1 equiv) in the presence of catalytic DMSO (Table 1, Entry 8) [27]; however, under these conditions, thiadiazine **1** gave only a trace of the desired ketone **3** (Table 1, Entry 8).

This study showed that the tetrachlorothiadiazine **1** and Appel’s salt **2** have similar reactivity to many of the investigated reagents, indicating that both compounds, despite having a different form (covalent *vs.* ionic) are similarly electrophilic. However, thiadiazine **1**, which is non-aromatic, is more sensitive to the presence of water in the reaction conditions.

### 2.3. Preparation of Ylidenemalononitriles ***10*** and ***11***

**Scheme 5:**
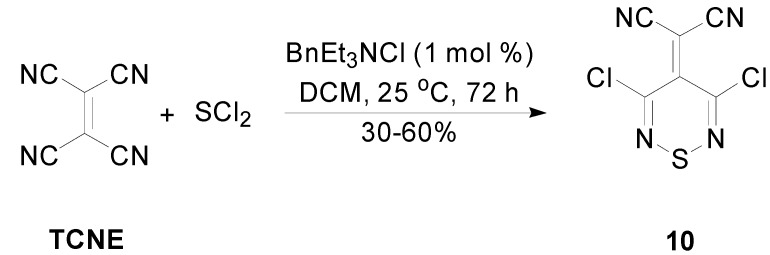
Preparation of the ylidenemalononitrile **10** from tetracyanoethene (TCNE) and SCl_2_.

The ylidenemalononitriles derived from tetrachlorothiadiazine **1**, and Appel’s salt **2**, 2-(3,5-dichloro-4*H*-1,2,6-thiadiazin-4-ylidene)malononitrile (**10**) and 2-(4-chloro-5*H*-1,2,3-dithiazol-5-ylidene)malononitrile (**11**), respectively, are both known and are useful heterocycles. The ylidenemalononitrile **10** has been used for the synthesis of other thiadiazine derivatives, such as furo- [28] and pyrrolo-fused thiadiazines [16,28,29] (Scheme 5), while (1,2,6-thiadiazinylidene)malononitriles are desired as possible electron acceptors for organic photovoltaic (OPV) devices [11,30]. Interestingly, the ylidenemalononitrile **10** could not be prepared from the Knoevenagel condensation of the thiadiazinone **3** with malononitrile, but was accessible from the reaction of expensive tetracyanoethene (TCNE) with SCl_2_. The reaction produced several minor side products and required chromatography to isolate the desired ylidene **10** in variable yields (30%–60%) [31]. These complications prevented scaling up the reaction and limited the supply of this useful ylidenemalononitrile.

**Scheme 6:**
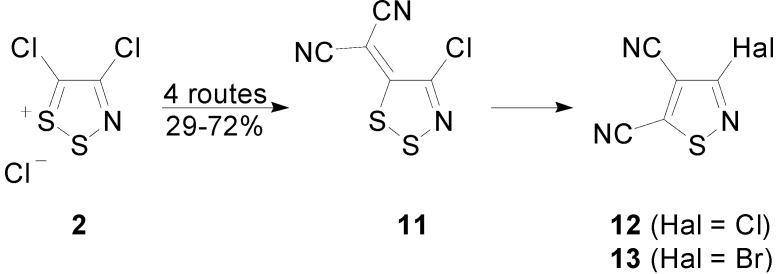
Synthesis of 2-(4-chloro-5*H*-1,2,3-dithiazol-5-ylidene)malononitrile (**11**) from Appel’s salt **2**. Structures of isothiazoles **12** and **13** derived from **11**.

2-(4-Chloro-5*H*-1,2,3-dithiazol-5-ylidene)malononitrile (**11**) is a useful starting material for difficult to access 3-chloroisothiazole-4,5-dicarbonitrile (**12**) [32,33] and 3-bromoisothiazole-4,5-dicarbonitrile (**13**) (Scheme 6) [33,34]. Its preparation starting from Appel’s salt **2** has been thoroughly investigated, and several syntheses have been reported, using malononitrile [32], tetracyanoethylene oxide (TCNEO) [32,35], halo-substituted malononitriles [34] and dimethylsulfonium dicyano-methylide [36].

**Scheme 7:**
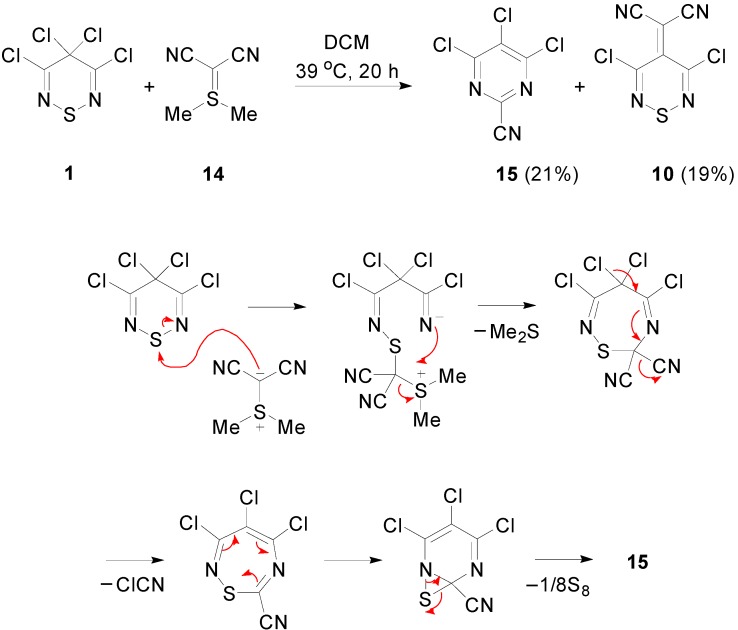
Reaction of tetrachlorothiadiazine **1** with dimethylsulfonium dicyanomethylide **14** and the proposed mechanism for the formation of the pyrimidine **15**.

In light of the similarities between the reactivity of tetrachlorothiadiazine **1** and Appel’s salt **2**, the transformation of **1** into the ylidenemalononitrile **10** was explored: while the reaction of Appel’s salt **2** with TCNEO (1 equiv) in PhMe heated at reflux gave the ylidene **11** in a 55% yield [32], under these reaction conditions, the tetrachlorothiadiazine **1** gave only a complex mixture of products containing a trace of the desired ylidenemalononitrile **10** by TLC. Similarly, while the reaction of dimethylsulfonium dicyanomethylide **14** with Appel’s salt **2** [36] affords the ylidene **11** in medium to good yields (29%–72%) along with other side products, the analogous reaction of tetrachlorothiadiazine **1** with dicyanomethylide **14** was less effective, giving only a low 19% yield of ylidene **10** as the only product. Interestingly, performing the reaction at *ca.* 39 °C gave surprisingly a mixture of 4,5,6-trichloropyrimidine-2-carbonitrile (**15**) and the ylidene **10** in 21% and 19% yields, respectively (Scheme 7). The formation of the pyrimidine **15** from this reaction was intriguing, as this heterocycle was first observed in the abovementioned reaction of TCNE with SCl_2_ [31]. A tentative mechanism for the formation of pyrimidine **15** is proposed below (Scheme 7).

Despite the fact that the simple pyridine-mediated condensation of malononitrile with Appel’s salt **2** gives only a low yield of ylidenemalononitrile **11** (40%) [32], when the tetrachlorothiadiazinone **1** was treated with malononitrile (1.1 equiv) and 2,6-lutidine (2 equiv) in dry DCM, at *ca.* 20 °C, the starting material was consumed quickly (TLC, 10 min) to give the ylidenemalononitrile **10** in a 78% yield (Table 2, Entry 1). Attempts to improve the yield of this reaction involved screening the base [1,8-diazabicyclo[5.4.0]undec-7-ene (DBU), *i*-Pr_2_NEt (Hünig’s base), K_2_CO_3_, *t-*BuOK or K_3_PO_4_] and solvents (MeCN, THF, PhMe), but these led to either degradation or to lower yields. Nevertheless, in DCM at *ca.* 20 °C using lutidine (2 equiv) as the base, increasing the equivalents of malononitrile from 1.1–1.5 equiv afforded the ylidenemalononitrile **10** in a slightly better yield (82%), while a further increase to 2 equiv of malononitrile gave a similar 83% yield (Table 2, Entries 2 and 3). The reaction could be scaled up to 4 mmol, and while this led to a drop in yield (73%) (Table 2, Entry 5), it also enabled a chromatography free work-up that involved passing the reaction mixture through a short plug of SiO_2_, washing the organic phase with 2M HCl and H_2_O and precipitating the product from THF/pentane to give the ylidene **10** in a preparatively useful 64% yield (Table 2, Entry 6).

**Table 2:** Transformation of the tetrachlorothiadiazine **1** into to the ylidenemalononitrile **10**. 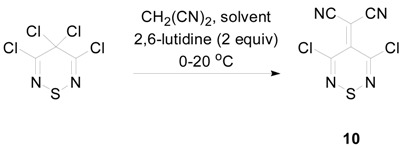

Entry	1 (mmol)	CH_2_(CN)_2_ (equiv)	DCM (mL)	Time (min)	Yield of 10 (%)
1	0.42	1.1	1	10	78
2	0.42	1.5	1	10	82
3	0.42	2	1	10	83
4	1	1.5	1	10	71
5	2	1.5	4	30	73
6	4	1.5	4	30	64 *^a^*

*^a^* Chromatography-free workup.

Attempts to develop a base-free protocol were less effective, as heating a PhMe solution of the tetrachlorothiadiazine **1** with malononitrile (2 equiv) at reflux (110 °C) led to complete consumption of the starting material only after 48 h and isolation of the desired ylidene **10** in a low 27% yield.

### 2.4. Cyclisation Reactions of 3,4,4,5-Tetrachloro-4H-1,2,6-thiadiazine *(**1**)*

**Scheme 8:**
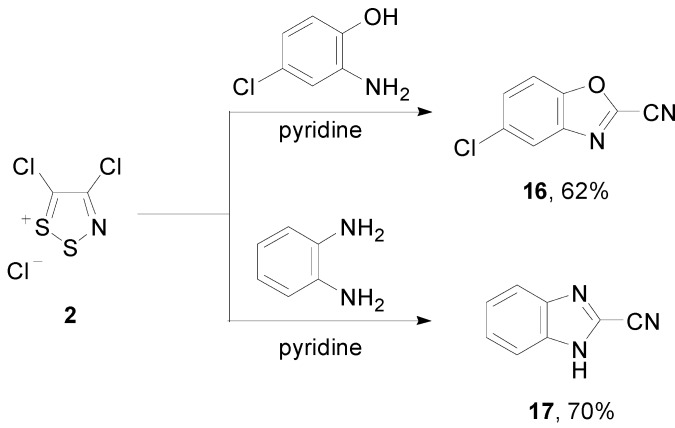
Reactions of Appel’s salt **2** with 2-amino-4-chlorophenol or benzene-1,2-diamine.

The condensation of Appel’s salt **2** with 2-amino-4-chlorophenol or benzene-1,2-diamine affords 5-chlorobenzo[*d*]oxazole-2-carbonitrile (**16**) and 1*H*-benzo[*d*]imidazole-2-carbonitrile (**17**), respectively (Scheme 8) [37].

**Scheme 9:**
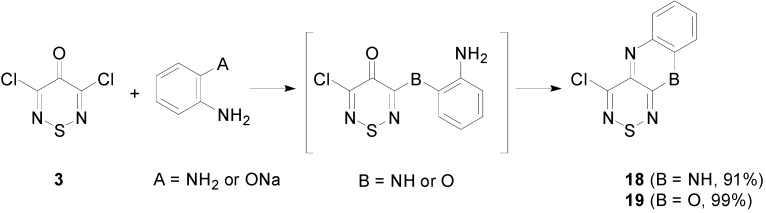
Reactions of thiadiazinone **3** with benzene-1,2-diamine and sodium 2-aminophenoxide.

Moreover, the condensation reactions of the thiadiazinone **3** with benzene-1,2-diamine or sodium 2-aminophenoxide to give fused systems are known (Scheme 9) [16]. Even though the thiadiazinone **3** was inert to reactions with primary amines at the C-4 position, after an initial nucleophilic addition in the C-3 position, intramolecular cyclizations readily occur with bisnucleophiles to afford tricyclic systems in excellent yields, e.g., 4-chloro-10*H*-[1,2,6]thiadiazino[3,4-*b*]quinoxaline (**18**) and 4-chloro-benzo[5,6][1,4]oxazino[2,3-*c*][1,2,6]thiadiazine (**19**) (Scheme 9). Worthy of note was that while an excellent yield was reported for oxazine **19**, this required an initial base activation (deprotonation) of the hydroxy group of 2-aminophenol to direct the reaction.

The dichloromethylene of the tetrachlorothiadiazine **1** was expected to be more electrophilic and therefore more reactive than the C-3, C-4 and C-5 positions of the thiadiazinone **3**. As such, bisnucleophiles were anticipated to initially attack the geminal dichloromethylene. This reactivity mimics that of Appel’s salt **2** (Scheme 8) with bisnucleophiles where the first nucleophilic displacement occurs at the more electrophilic C-5 position.

**Scheme 10:**
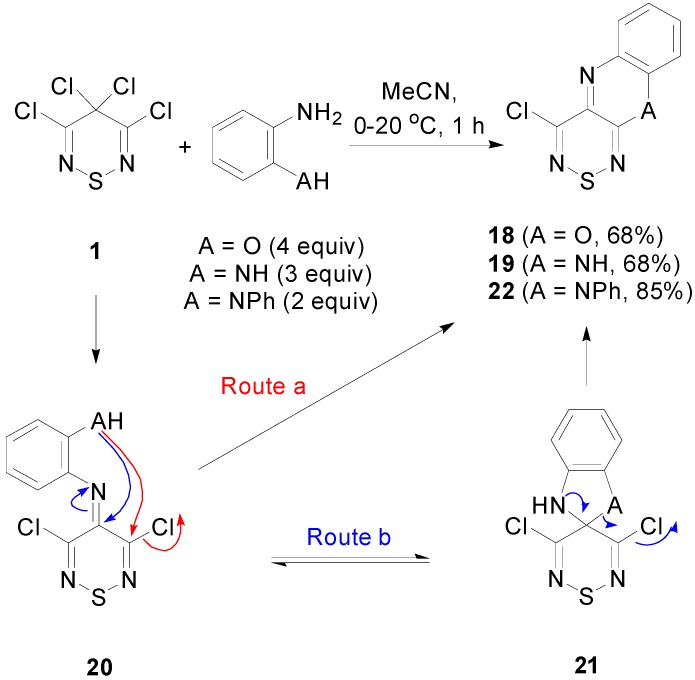
Reactions of the tetrachlorothiadiazine **1** with 2-aminophenol, benzene-1,2-diamine and *N*′*-*phenylbenzene-1,2-diamine.

Treatment of the tetrachlorothiadiazine **1** with 2-aminophenol or benzene-1,2-diamine in MeCN at 20 °C for 1 h afforded the fused heterocycles **18** (68%) and **19** (68%), respectively (Scheme 10). Tentatively, the moderate yields (68%) can be attributed to the high reactivity of the tetrachlorothiadiazine **1** that can presumably suffer from both halophilic and thiophilic attack, leading to its degradation.

A proposed mechanism for this transformation involves the initial formation of imine **20**, which we know from previous studies readily forms by reaction of thiadiazine **1** with arylamines [22]. Subsequently, two routes are proposed, either a 6-exo cyclisation to give the final products (Route a) or a 5-endo cyclisation occurs to form the spirocyclic compounds **21** that then ring-opens, assisted by electron release from both the nitrogen’s lone pair and possibly the ring sulfur, subsequently cyclizing at the C-3 position (route b). Interestingly, when using *N*′*-*phenylbenzene-1,2-diamine, an unsymmetrical diamine, the less reactive secondary amine ends up cyclizing on the thiadiazine C-3 position to give 4-chloro-10-phenyl-10*H*-[1,2,6][3,4-*b*]quinoxaline (**22**) in an 85% yield. While this route to tricyclic systems **18**, **19** and **22** was non-quantitative, it offered two distinct advantages: firstly, it avoided the need to access the thiadiazinone **3**, thereby reducing the number of steps to the final products, and secondly, it offered an alternative regioselectivity that avoided the need to base activate bisnucleophiles, such as the 2-aminophenol.

### 2.5. Reactivity of 4-Chloro-10H-[1,2,6]thiadiazino[3,4-b]quinoxaline *(**19**)*

**Scheme 11:**
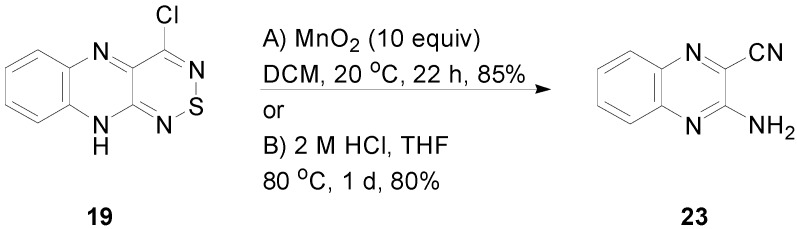
Conversion of 4-chloro-10*H*-[1,2,6]thiadiazino[3,4-*b*]quinoxaline (**19**) to 3-aminoquinoxaline-2-carbonitrile (**23**).

Interestingly, during the synthesis and isolation of 4-chloro-10*H*-[1,2,6]thiadiazino[3,4-*b*]quinoxaline (**19**), we observed its decomposition to 3-aminoquinoxaline-2-carbonitrile (**23**). Traces of this product were initially observed during chromatography of quinoxaline **19**, so we decided to investigate the reactivity of this compound. Quinoxaline **19** was stable under basic conditions, as it was recovered unchanged after 48 h stirring in neat Et_3_N, while it was unstable in acid, as heating a solution in glacial AcOH at reflux for 15 min led to complete consumption of the starting material and isolation of 3-aminoquinoxaline-2-carbonitrile (**23**) as the only product in a 38% yield. Alternatively, heating a solution of **19** in aqueous HCl/THF at 80 °C gave the quinoxaline **23** in an 80% yield (Scheme 11).

Furthermore, quinoxaline **19** was also unstable in oxidizing and reducing conditions. Namely, stirring a DCM solution of **19** with MnO_2_ (10 equiv) at *ca.* 20 °C led to complete consumption of the starting material after 22 h and isolation of aminoquinazoline **23** in an 85% yield (Scheme 11). The dissolving metal reduction with Zn (4 equiv) in AcOH led to degradation of **19** and isolation of 3-aminoquinoxaline-2-carbonitrile (**23**) in only a low 6% yield.

Aminoquinoxaline **23** was previously prepared in a low yield by the reaction of 4-chloro-5-cyano-1,2,3-dithiazolium chloride with benzene-1,2-diamine [38]. Similar degradation reactions were also investigated for thiadiazines **18** and **22**; however, these gave complex mixtures of products (by TLC) and were not pursued further.

## 3. Experimental Section

### 3.1. General Methods and Materials

All chemicals were commercially available, except those whose synthesis is described. Anhydrous Na_2_SO_4_ was used for drying organic extracts, and all volatiles were removed under reduced pressure. All reaction mixtures and column eluents were monitored by TLC using commercial glass-backed thin layer chromatography (TLC) plates (Merck Kieselgel 60 F_254_) [39]. The plates were observed under UV light at 254 and 365 nm. The technique of dry flash chromatography was used throughout for all non-TLC-scale chromatographic separations using Merck Silica Gel 60 (less than 0.063 mm, Merck KGaA, Darmstadt, Germany). Melting points were determined using a PolyTherm-A, Wagner & Munz, Kofler—Hotstage Microscope apparatus (Wagner & Munz, Munich, Germany) or were determined using a TA Instruments differential scanning calorimeter (DSC) Q1000 with samples hermetically sealed in aluminum pans under an argon atmosphere; using heating rates of 5 °C/min (DSC melting points listed by onset and peak values). Solvents used for recrystallization are indicated after the melting point. UV spectra were obtained using a Perkin-Elmer Lambda-25 UV-VIS spectrophotometer (Perkin-Elmer, Waltham, MA, USA), and inflections are identified by the abbreviation “inf”. IR spectra were recorded on a Shimadzu FTIR-NIR Prestige-21 spectrometer (Shimadzu, Kyoto, Japan) with the Pike Miracle Ge ATR accessory (Pike Miracle, Madison, WI, USA), and strong, medium and weak peaks are represented by s, m and w, respectively. ^1^H- and ^13^C-NMR spectra were recorded on a Bruker Avance 300 (at 300 and 75 MHz, respectively, Bruker, Billerica, MA, USA) or a 500 machine (at 500 and 125 MHz, respectively). Deuterated solvents were used for the homonuclear lock, and the signals are referenced to the deuterated solvent peaks. Attached-proton-test (APT) NMR studies identified quaternary and tertiary carbons, which are indicated by (s) and (d) notations, respectively. MALDI-TOF mass spectra were recorded on a Bruker Autoflex III Smartbeam instrument. Low resolution (EI) mass spectra were recorded on a Shimadzu Q2010 GC-MS with a direct inlet probe. Dichloromalononitrile (**6**) [24], *N*-2,2-trichloro-2-cyanoacetimidoyl chloride (**7**) [24] and dimethylsulfonium dicyanomethylide (**14**) [40] were prepared according to the reported procedures.

### 3.2. Preparation of 3,4,4,5-Tetrachloro-4H-1,2,6-thiadiazine *(**1**)*

#### 3.2.1. Reaction of Dichloromalononitrile with SCl_2_

Performed according to the literature procedure [26]. To a stirred solution of dichloromalononitrile (**6**) (100 g, 0.740 mol) was added BnEt_3_NCl (1.69 g, 7.40 mmol), and the mixture was cooled in an ice bath (0 °C). Freshly distilled SCl_2_ (76.2 g, 0.740 mol) was then added dropwise, the cooling bath removed and the mixture stirred overnight, protected with a CaCl_2_ drying tube. After 18 h, the solvent was evaporated under vacuum and the product distilled under reduced pressure (90 °C, 30 mbar) to give 3,4,4,5-tetrachloro-4*H*-1,2,6-thiadiazine (**1**) (121.5 g, 69%) as a pale yellow oil that crystallized on cooling to −20 °C mp (DSC) onset: 10.3 °C, peak max: 12.8 °C; *δ*_C_ (125 MHz; CDCl_3_) 138.5 (s), 72.1 (s); ν_max_/cm^−1^ 1582m, 1566m, 1144s, 1030s, 953m, 812m, 770s; identical to an authentic sample.

#### 3.2.2. Reaction of *N*-2,2-Trichloro-2-cyanoacetimidoyl Chloride (**4**) with S_8_

This was performed according to the literature procedure [26]. A stirred solution of *N*-2,2-trichloro-2-cyanoacetimidoyl chloride (**7**) (41.2 g, 0.200 mol) in dry DCM (40 mL) was cooled in an ice bath (0 °C), and BnEt_3_NCl (456 mg, 2.00 mmol) was added, followed by S_8_ (6.40 g, 25.0 mmol) added in one portion. The cooling bath was then removed and the mixture stirred overnight, protected with a CaCl_2_ drying tube. After 18 h, the product was distilled under reduced pressure (90 °C, 30 mbar) to give 3,4,4,5-tetrachloro-4*H*-1,2,6-thiadiazine (**1**) (36.2 g, 76%) as a pale yellow oil that crystallized on cooling to −20 °C mp (DSC) onset: 10.3 °C, peak max: 12.8 °C; identical to that described above.

### 3.3. Transformations of 3,4,4,5-Tetrachloro-4H-1,2,6-thiadiazine *(**1**)* to 3,5-Dichloro-4H-1,2,6-thiadiazin-4-one *(**3**)*

#### 3.3.1. Reaction of 3,4,4,5-Tetrachloro-4*H*-1,2,6-thiadiazine (**1**) with Formic Acid

This was performed according to the literature procedure [15]. 3,4,4,5-Tetrachloro-4*H*-1,2,6-thiadiazine (**1**) (59.5 g, 0.250 mol) was added dropwise to a cold (10 °C) stirred solution of 98% HCO_2_H (175 mL). The cooling bath was then removed and the solution stirred overnight, protected with a CaCl_2_ drying tube. After complete consumption of the starting material (TLC, 24 h), the mixture was poured into ice water and the precipitate collected, washed with water and dried under air to yield 3,5-dichloro-4*H*-1,2,6-thiadiazin-4-one (**3**) (34.3 g, 75%) as yellowish needles, mp 81–82 °C (from *c*-hexane, lit. [15] 82–83 °C); R*_f_* 0.56 (*n*-hexane/DCM, 50:50); *δ*_C_ (125 MHz; CDCl_3_) 157.5 (s), 149.8 (s); ν_max_/cm^−1^ 1657s (C=O), 1501m, 1277w, 1265w, 1248m, 1227m, 1065s, 854m, 847w, 745s, identical to an authentic sample [15].

#### 3.3.2. Reaction of 3,4,4,5-Tetrachloro-4*H*-1,2,6-thiadiazine (**1**) with Acetic Acid

3,4,4,5-Tetrachloro-4*H*-1,2,6-thiadiazine (**1**) (238 mg, 1.00 mmol) was added dropwise to a stirred solution of glacial AcOH (1 mL), and the solution was stirred at *ca.* 20 °C, protected with a CaCl_2_ drying tube, until complete consumption of the starting material (TLC, 48 h). The mixture was then adsorbed onto silica, and chromatography (*n*-hexane/DCM, 50:50) gave 3,5-dichloro-4*H*-1,2,6-thiadiazin-4-one (**3**) (135 mg, 74%) as yellowish needles, mp 81–82 °C (from *c*-hexane, lit. [15] 82–83 °C), identical to that described above.

#### 3.3.3. Reaction of 3,4,4,5-Tetrachloro-4*H*-1,2,6-thiadiazine (**1**) with NaNO_3_ in MeCN

To a solution of 3,4,4,5-tetrachloro-4*H*-1,2,6-thiadiazine (**1**) (100 mg, 0.42 mmol) in dry MeCN (1 mL) was added NaNO_3_ (39 mg, 0.46 mmol), and the mixture was stirred at *ca.* 20 °C, protected with a CaCl_2_ drying tube, until consumption of the starting material (TLC, 1.5 h). The reaction mixture was then adsorbed onto silica, and chromatography (*n*-hexane/DCM, 50:50) gave 3,5-dichloro-4*H*-1,2,6-thiadiazin-4-one (**3**) (55 mg, 71%) as yellowish needles, mp 81–82 °C (from *c*-hexane, lit. [15] 82–83 °C), identical to that described above.

#### 3.3.4. Reaction of 3,4,4,5-Tetrachloro-4*H*-1,2,6-thiadiazine (**1**) with AgNO_3_ in MeCN

To a solution of 3,4,4,5-tetrachloro-4*H*-1,2,6-thiadiazine (**1**) (100 mg, 0.42 mmol) in dry MeCN (1 mL) was added AgNO_3_ (71 mg, 0.42 mmol), and the mixture was stirred at *ca.* 20 °C, protected with a CaCl_2_ drying tube, until consumption of the starting material (TLC, 30 min). A colorless precipitate formed that was filtered to give AgCl (52 mg, 87%). The filtrate was then adsorbed onto silica, and chromatography (*n*-hexane/DCM, 50:50) gave 3,5-dichloro-4*H*-1,2,6-thiadiazin-4-one (**3**) (65 mg, 85%) as yellowish needles, mp 81–82 °C (from *c*-hexane, lit. [15] 82–83 °C), identical to that described above.

#### 3.3.5. Reaction of 3,4,4,5-Tetrachloro-4*H*-1,2,6-thiadiazine (**1**) with DMSO in MeCN

To a solution of 3,4,4,5-tetrachloro-4*H*-1,2,6-thiadiazine (**1**) (238 mg, 1.00 mmol) in dry MeCN (10 mL) was added DMSO (71 μL, 1.0 mmol), and the mixture was stirred at *ca.* 20 °C, protected with a CaCl_2_ drying tube, until consumption of the starting material (TLC, 20 h). The reaction mixture was then adsorbed onto silica, and chromatography (*n*-hexane/DCM, 50:50) gave 3,5-dichloro-4*H*-1,2,6-thiadiazin-4-one (**3**) (57 mg, 31%) as yellowish needles, mp 81–82 °C (from *c*-hexane, lit. [15] 82–83 °C), identical to the one reported above.

#### 3.3.6. Reaction of 3,4,4,5-Tetrachloro-4*H*-1,2,6-thiadiazine (**1**) with Neat DMSO

A solution of 3,4,4,5-tetrachloro-4*H*-1,2,6-thiadiazine (**1**) (238 mg, 1.00 mmol) in DMSO (2 mL) was stirred at *ca.* 20 °C, protected with a CaCl_2_ drying tube, until consumption of the starting material (TLC, 1 h). Water (20 mL) was then added and the mixture extracted with *t*-BuOMe (3 × 20 mL), the organic phase combined, dried (Na_2_SO_4_) and evaporated to give crude **3**. The crude product was then adsorbed onto silica, and chromatography (*n*-hexane/DCM, 50:50) gave 3,5-dichloro-4*H*-1,2,6-thiadiazin-4-one (**3**) (82 mg, 45%) as yellowish needles, mp 81–82 °C (from *c*-hexane, lit. [15] 82–83 °C), identical to the one reported above.

### 3.4. Transformations of 4,5-Dichloro-1,2,3-dithiazolium Chloride *(**2**)* into 4-Chloro-5H-1,2,3-dithiazol-5-one *(**9**)*

#### 3.4.1. Reaction of 4,5-Dichloro-1,2,3-dithiazolium Chloride (**2**) with Formic Acid

Appel’s salt **2** (208 mg, 1.00 mmol) was added to a stirred solution of HCO_2_H (98%, 1 mL), and the solution was stirred at *ca.* 20 °C, protected with a CaCl_2_ drying tube, until complete consumption of the starting material (2 h). The mixture was then adsorbed onto silica, and chromatography (*n*-hexane/DCM, 60:40) gave 4-chloro-5*H*-1,2,3-dithiazol-5-one (**9**) (137 mg, 89%) as pale yellow plates, mp 35–36 °C (from pentane, lit. [17] 39 °C); R*_f_* 0.48 (*n*-hexane/DCM, 60:40); *δ*_C_ (75 MHz; CDCl_3_) 183.3 (s), 147.0 (s); ν_max_/cm^−1^ 1651s (C=O), 1612m, 1501m, 1337w, 1142w, 1021m, 1080w, 847m, 837m, 806w, 785m, identical to an authentic sample [17].

#### 3.4.2. Reaction of 4,5-Dichloro-1,2,3-dithiazolium Chloride (**2**) with Acetic Acid

Appel’s salt **2** (208 mg, 1.00 mmol) was added to a stirred solution of glacial AcOH (1 mL), and the solution was stirred at *ca.* 20 °C, protected with a CaCl_2_ drying tube, until complete consumption of the starting material (2 h). The mixture was then adsorbed onto silica, and chromatography (*n*-hexane/DCM, 60:40) gave 4-chloro-5*H*-1,2,3-dithiazol-5-one (**9**) (92 mg, 60%) as pale yellow plates, mp 35–36 °C (from pentane, lit. [17] 39 °C), identical to that described above.

#### 3.4.3. Reaction of 4,5-Dichloro-1,2,3-dithiazolium Chloride (**2**) with NaNO_3_ in MeCN

To a suspension of Appel’s salt (**2**) (104 mg, 0.50 mmol) in dry MeCN (1 mL) was added NaNO_3_ (43 mg, 0.50 mmol), and the mixture was stirred at *ca.* 20 °C, protected with a CaCl_2_ drying tube, until consumption of the starting material (18 h). The mixture was then adsorbed onto silica, and chromatography (*n*-hexane/DCM, 60:40) gave 4-chloro-5*H*-1,2,3-dithiazol-5-one (**9**) (59 mg, 77%) as pale yellow plates, mp 35–36 °C (from pentane, lit. [17] 39 °C), identical to that described above.

#### 3.4.4. Reaction of 4,5-Dichloro-1,2,3-dithiazolium Chloride (**2**) with AgNO_3_ in MeCN

To a suspension of Appel’s salt (**2**) (104 mg, 0.50 mmol) in dry MeCN (1 mL) was added AgNO_3_ (85 mg, 0.50 mmol), and the mixture was stirred at *ca.* 20 °C, protected with a CaCl_2_ drying tube, until consumption of the starting material (1 min). A colorless precipitate formed that was filtered to give AgCl (72 mg, 100%). The filtrate was then adsorbed onto silica and chromatography (*n*-hexane/DCM, 60:40) gave 4-chloro-5*H*-1,2,3-dithiazol-5-one (**9**) (55 mg, 72%) as pale yellow plates, mp 35–36 °C (from pentane, lit. [17] 39 °C), identical to that described above.

### 3.5. Transformations of 3,4,4,5-Tetrachloro-4H-1,2,6-thiadiazine *(**1**)* to 2-(3,5-Dichloro-4H-1,2,6-thiadiazin-4-ylidene)malononitrile *(**10**)*

#### 3.5.1. Reaction of 3,4,4,5-Tetrachloro-4*H*-1,2,6-thiadiazine (**1**) with Malononitrile

To a solution of 3,4,4,5-tetrachloro-4*H*-1,2,6-thiadiazine (**1**) (100 mg, 0.42 mmol) in dry DCM (1 mL) was added malononitrile (42 mg, 0.63 mmol), and the solution was cooled in an ice bath (0 °C). 2,6-Lutidine (98 μL, 0.84 mmol) was then added dropwise to the stirred solution. The cooling bath was then removed and the solution stirred at *ca.* 20 °C until complete consumption of the starting material (TLC, 10 min). The reaction mixture was then poured onto a silica column, and chromatography (*n*-hexane/DCM, 20:80) gave 2-(3,5-dichloro-4*H*-1,2,6-thiadiazin-4-ylidene)malononitrile (**10**) (80 mg, 82%) as yellow plates, mp 133–134 °C (from *c*-hexane, lit. [31] 134–135 °C); R*_f_* 0.65 (*n*-hexane/DCM, 20:80); *δ*_C_ (125 MHz; CDCl_3_) 138.8 (s), 136.5 (s), 112.0 (s), 81.2 (s); ν_max_/cm^−1^ 2216m (C≡N), 1522m, 1508m, 1487m, 1288s, 1273m, 1144m, 1082m, 812m, 754s; *m*/*z* (EI) 230 (M^+^, 100), 204 (M^+^-CN, 2%), 195 (M^+^-Cl, 55), 169 (M^+^-CClN, 21), 134 (M^+^-CCl_2_N, 21), identical to an authentic sample [31].

#### 3.5.2. Reaction of 3,4,4,5-Tetrachloro-4*H*-1,2,6-thiadiazine (**1**) with Malononitrile, Chromatography-Free (Table 2, Entry 6)

To a solution of 3,4,4,5-tetrachloro-4*H*-1,2,6-thiadiazine (**1**) (952 mg, 4.00 mmol) in dry DCM (4 mL) was added malononitrile (396 mg, 6.00 mmol), and the solution was cooled in an ice bath (0 °C). 2,6-Lutidine (0.930 mL, 8.00 mmol) was then added dropwise to the stirred solution. The cooling bath then removed and the solution stirred at *ca.* 20 °C until complete consumption of the starting material (TLC, 30 min). The reaction mixture was then passed through a plug of silica (DCM) and washed with 2 M HCl (2 × 10 mL) and H_2_O (10 mL), evaporated and precipitated from THF/pentane to give 2-(3,5-dichloro-4*H*-1,2,6-thiadiazin-4-ylidene)malononitrile (**10**) (591 mg, 64%) as yellow plates, mp 133–134 °C (from *c*-hexane, lit. [31] 134–135 °C), identical to that described above.

#### 3.5.3. Reaction of 3,4,4,5-Tetrachloro-4*H*-1,2,6-thiadiazine (**1**) with Dimethylsulfonium Dicyanomethylide (**14**)

To a solution of 3,4,4,5-tetrachloro-4*H*-1,2,6-thiadiazine (**1**) (100 mg, 0.42 mmol) in dry DCM (1 mL) was added dimethylsulfonium dicyanomethylide (**14**) (58 mg, 0.46 mmol), and the mixture was heated at *ca.* 39 °C, protected with a CaCl_2_ drying tube, until consumption of the starting material (20 h). The reaction mixture was then adsorbed onto silica, and chromatography (*n*-hexane/DCM, 60:40) gave 4,5,6-trichloropyrimidine-2-carbonitrile (**15**) (18 mg, 21%) as colorless needles, mp 62–63 °C (sublimation, lit. [31] 64–65 °C); R*_f_* 0.41 (*n*-hexane/DCM, 60:40); ν_max_/cm^−1^ 2423w and 2363w (C≡N) and 1530w (C=C), 1497s, 1350s, 1337m, 1315m, 1300m, 1275m, 1256m, 1209m, 1065m, 1057m, 910m, 832m, 818m, 770m; *δ*_C_ (125 MHz; CDCl_3_) 161.1 (s), 139.7 (s), 133.2 (s), 113.4 (s); *m*/*z* (EI) 207 (M^+^, 100), 172 (M^+^-Cl, 50%), 120 (C_3_Cl_2_N^+^, 28), 111 (C_4_ClN_2_^+^, 13), identical to an authentic sample [31]. Further elution (*n*-hexane/DCM, 20:80) gave 2-(3,5-dichloro-4*H*-1,2,6-thiadiazin-4-ylidene)malononitrile (**10**) (18 mg, 19%) as yellow plates, mp 133–134 °C (from *c*-hexane, lit. [31] 134–135 °C), identical to that described above.

### 3.6. Cyclisation Reactions of 3,4,4,5-Tetrachloro-4H-1,2,6-thiadiazine (**1**)

#### 3.6.1. Synthesis of 4-Chlorobenzo[5,6][1,4]oxazino[2,3-*c*][1,2,6]thiadiazine (**18**) (General Procedure)

To a cold (0 °C) stirred solution of tetrachlorothiadiazine **1** (238 mg, 1.00 mmol) in dry MeCN (5 mL) was added in one portion 2-aminophenol (436 mg, 4.00 mmol). The cooling bath was then removed and the solution stirred at *ca.* 20 °C protected with a CaCl_2_ drying tube until complete consumption of the starting material (TLC, 1 h). The mixture was then adsorbed onto silica, and chromatography (*n*-hexane/DCM, 50:50) gave the title compound **18** (179 mg, 76%) as orange prisms, mp 157–160 °C (from EtOH, lit. [16] 158–163 °C); R*_f_* 0.43 (*n*-hexane/DCM, 50:50); ν_max_/cm^−1^ 3103w and 3048w (Ar CH), 1614m, 1587m, 1547m, 1518m, 1456s, 1365m, 1331s, 1308m, 1294w, 1283m, 1244m, 1206m, 1184m, 1107m, 1059m, 1026m, 978m, 968m, 945m, 901s, 876s, 868m, 800s, 763s, 748s; *δ*_H_ (500 MHz; DMSO-*d*_6_) 7.24 (1H, dd, *J* = 7.7, 1.4, Ar *H*), 7.18 (1H, ddd, *J* = 7.8, 7.8, 1.5, Ar *H*), 7.05 (1H, ddd, *J =* 7.6, 7.6, 0.9, Ar *H*), 6.89 (1H, d, *J* = 8.1, Ar *H*); *δ*_C_ (125 MHz; DMSO-*d*_6_) 151.0 (s), 148.1 (s), 145.8 (s), 138.3 (s), 133.9 (s), 130.6 (d), 128.5 (d), 125.7 (d), 115.2 (d); *m*/*z* (EI) 237 (M^+^, 100%), 202 (M^+^-Cl, 5), 176 (M^+^-CClN, 9), 144 (M^+^-CClNS, 11), 118 (5), 93 (CClNS^+^, 11), 64 (6), identical to an authentic sample.

#### 3.6.2. Synthesis of 4-Chloro-10*H*-[1,2,6]thiadiazino[3,4-*b*]quinoxaline (**19**)

Similar treatment of tetrachlorothiadiazine **1** (238 mg, 1.00 mmol) with benzene-1,2-diamine (432 mg, 3.00 mmol) gave, after the solvent was evaporated *in vacuo* and the crude product precipitated from EtOH (2 mL), the title compound **19** (160 mg, 68%) as purple needles, mp 310 °C (from EtOH, lit. [16] 310 °C subl.); R*_f_* 0.17 (*n*-hexane/DCM, 50:50); ν_max_/cm^−1^ 3237w, 3188w, 3140w, 3069w and 3051w (Ar CH), 1605s, 1570w, 1514m, 1470m, 1414m, 1379s, 1275m, 1221m, 1115m, 957m, 924s, 878m, 800s, 766s, 741s; *δ*_H_ (500 MHz; DMSO-*d*_6_) 10.19 (1H, s, N*H*), 6.93 (1H, ddd, *J* = 7.8, 7.8, 1.1, Ar *H*), 6.87 (1H, d, *J* = 8.2, Ar *H*), 6.66 (1H, ddd, *J =* 7.9, 7.9, 0.8, Ar *H*), 6.38 (1H, d, *J* = 7.9, Ar *H*); *δ*_C_ (125 MHz; DMSO-*d*_6_) 149.9 (s), 142.2 (s), 139.7 (s), 136.4 (s), 135.8 (s), 130.5 (d), 128.3 (d), 123.1 (d), 113.5 (d); *m*/*z* (EI) 236 (M^+^, 100%), 201 (M^+^-Cl, 32), 174 (M^+^-CHClN, 8), 168 (7), 162 (4), 154 (6), 149 (6), 143 (M^+^-CClNS, 21), 131 (6), 118 (6), 102 (5), 90 (9), 76 (C_6_H_4_^+^,4), 69 (11), identical to the an authentic sample.

#### 3.6.3. Synthesis of 4-Chloro-10-phenyl-10*H*-[1,2,6][3,4-*b*]quinoxaline (**22**)

Similar treatment of tetrachlorothiadiazine **1** (238 mg, 1.00 mmol) with *N*′-phenylbenzene-1,2-diamine (368 mg, 2.00 mmol) gave after chromatography (DCM) the title compound **22** (212 mg, 68%) as brown needles, mp 256–258 °C (from PhMe); R*_f_* 0.81 (DCM); (found: C, 57.26; H, 2.54; N, 17.62. C_15_H_9_ClN_4_S requires C, 57.60; H, 2.90; N, 17.91%); λ_max_ (DCM)/nm 267 (log *ε* 4.37), 285 inf (4.29), 296 inf (4.24), 326 (4.14), 367 (3.94), 387 (3.98), 409 (3.83), 527 (3.68), 552 (3.69), 596 inf (3.44); ν_max_/cm^−1^ 3048w, 1593m, 1504m, 1497m, 1485m, 1450m, 1360s, 1317m, 1173w, 1157m, 941m, 932m, 876m, 764s, 750s, 731s, 714s; *δ*_H_ (500 MHz; DMSO-*d*_6_) 7.58 (2H, dd, *J* = 7.4, 7.4, Ar *H*), 7.47 (1H, dd, *J* = 7.3, 7.3, Ar *H*), 7.33 (2H, d, *J* = 7.4, Ar *H*), 7.06 (1H, d, *J* = 7.5, Ar *H*), 6.87 (1H, dd, *J* = 7.4, 7.4, Ar *H*), 6.79 (1H, dd, *J* = 7.4, 7.4, Ar *H*), 5.66 (1H, d, *J* = 7.9, Ar *H*); *δ*_C_ (125 MHz; DMSO-*d*_6_) 149.6 (s), 143.3 (s), 139.3 (s), 138.4 (s), 135.7 (s), 135.2 (s), 130.6 (d), 130.3 (d), 129.0 (d), 128.8 (d), 128.7 (d), 123.7 (d), 113.5 (d); *m*/*z* (MALDI-TOF) 314 (M^+^+2, 47%), 312 (M^+^, 95), 277 (M^+^-Cl, 100), 245 (M^+^-ClS, 36).

### 3.7. Reactivity of 4-Chloro-10H-[1,2,6]thiadiazino[3,4-b]quinoxaline *(**19**)*

#### 3.7.1. Reaction of 4-Chloro-10*H*-[1,2,6]thiadiazino[3,4-*b*]quinoxaline (**19**) with MnO_2_

To a stirred solution of 4-chloro-10*H*-[1,2,6]thiadiazino[3,4-*b*]quinoxaline (19) (47 mg, 0.20 mmol) in DCM (2 mL) at *ca.* 20 °C was added MnO_2_ (174 mg, 2.0 mmol), and the mixture was protected with a CaCl_2_ drying tube until complete consumption of the starting material (TLC, 22 h). The solid that formed was removed by filtration and the filtrate adsorbed onto silica and chromatographed (*n*-hexane/*t-*BuOMe, 40:60) to give 3-aminoquinoxaline-2-carbonitrile (**23**) (29 mg, 85%) as yellow needles, mp 209–210 °C (from *c*-hexane, lit. [38] 210 °C); R*_f_* 0.59 (*n*-hexane/*t-*BuOMe, 40:60); ν_max_/cm^−1^ 3412m, 3327m, 3132 br, 2232m (C≡N), 1661s, 1611m, 1562m, 1557m, 1489m, 1437m, 1371m, 1360m, 1323w, 1254w, 1223m, 1171w, 1144m, 1123w, 1092w, 1013w, 963w, 918m, 760s, 754s, 741m; *δ*_H_ (500 MHz; CDCl_3_) 7.94 (1H, dd, *J* = 8.4, 1.0, Ar *H*), 7.74 (1H, ddd, *J* = 8.4, 6.8, 1.4, Ar *H*), 7.69 (1H, dd, *J* = 8.5, 1.1, Ar *H*), 7.53 (1H, ddd, *J* = 8.3, 6.8, 1.4, Ar *H*), 5.42 (2H, br s, N*H*_2_); *δ*_C_ (125 MHz; CDCl_3_) 151.8 (s), 142.5 (s), 137.5 (s), 133.8 (d), 129.7 (d), 126.8 (d), 126.4 (d), 119.2 (s), 114.9 (s); *m*/*z* (EI) 170 (M^+^, 100%), 143 (M^+^-CHN, 33), 118 (17), 91 (C_6_H_5_N^+^, 13), 84 (13), 76 (C_6_H_4_^+^, 6), 66 (13), identical to an authentic sample [38].

#### 3.7.2. Reaction of 4-Chloro-10*H*-[1,2,6]thiadiazino[3,4-*b*]quinoxaline (**19**) with HCl

To a stirred solution of 4-chloro-10*H*-[1,2,6]thiadiazino[3,4-*b*]quinoxaline (**19**) (47 mg, 0.20 mmol) in THF (4 mL) was added a 2 M aqueous HCl (4.0 mL, 8.0 mmol), and the mixture was stirred at *ca.* 80 °C until complete consumption of the starting material (TLC, 24 h). The mixture was then cooled to *ca.* 20 °C, and DCM (10 mL) was added, followed by saturated Na_2_CO_3_ until the pH reached 10. The organic phase was separated and the mixture further extracted with DCM (2 × 10 mL), and the combined organic phase was then dried (Na_2_SO_4_) and evaporated. The crude product was then adsorbed onto silica and chromatographed (*n*-hexane/*t-*BuOMe, 40:60) to give 3-aminoquinoxaline-2-carbonitrile (**23**) (27 mg, 80%) as yellow needles, mp 209–210 °C (from *c*-hexane, lit. [38] 210 °C); R*_f_* 0.59 (*n*-hexane/*t-*BuOMe, 40:60), identical to that described above.

## 4. Conclusions

Three modes of reactivity were investigated for the sparingly explored 3,4,4,5-tetrachloro-4*H*-1,2,6-thiadiazine (**1**): its conversion to the useful scaffolds 3,5-dichloro-4*H*-1,2,6-thiadiazin-4-one (**3**) and 2-(3,5-dichloro-4*H*-1,2,6-thiadiazin-4-ylidene)malononitrile (**10**) in 85% and 83% yields, respectively, and its transformation to tricyclic thiadiazines in good yields. The geminal dichloromethylene to ketone transformation was directly compared between thiadiazine **1** and Appel’s salt **2**, and similarities and differences between the reactivity of the two reagents were identified. The reaction of thiadiazine **1** with dicyanomethylide **14** led to the isolation of an unexpected side product, 4,5,6-trichloropyrimidine-2-carbonitrile (**15**), the investigation of which is now under further study. The development of the chemistry of 3,4,4,5-tetrachloro-4*H*-1,2,6-thiadiazine (**1**) shows that this unexplored reagent can be a useful scaffold in the synthesis of valuable heterocycles.
